# Augmenting Multi-Instance Multilabel Learning with Sparse Bayesian Models for Skin Biopsy Image Analysis

**DOI:** 10.1155/2014/305629

**Published:** 2014-04-07

**Authors:** Gang Zhang, Jian Yin, Xiangyang Su, Yongjing Huang, Yingrong Lao, Zhaohui Liang, Shanxing Ou, Honglai Zhang

**Affiliations:** ^1^School of Information Science and Technology, Sun Yat-Sen University, Guangzhou 510275, China; ^2^School of Automation, Guangdong University of Technology, Guangzhou 510006, China; ^3^Department of Dermatology and Venerology, The 3rd Affiliated Hospital of Sun Yat-Sen University, Guangzhou 510630, China; ^4^The 2nd Affiliated Hospital, Guangzhou University of Chinese Medicine, Guangzhou 510405, China; ^5^Department of Radiology, Guangzhou General Hospital of Guangzhou Military Command, Guangzhou 510010, China

## Abstract

Skin biopsy images can reveal causes and severity of many skin diseases, which is a significant complement for skin surface inspection. Automatic annotation of skin biopsy image is an important problem for increasing efficiency and reducing the subjectiveness in diagnosis. However it is challenging particularly when there exists indirect relationship between annotation terms and local regions of a biopsy image, as well as local structures with different textures. In this paper, a novel method based on a recent proposed machine learning model, named multi-instance multilabel (MIML), is proposed to model the potential knowledge and experience of doctors on skin biopsy image annotation. We first show that the problem of skin biopsy image annotation can naturally be expressed as a MIML problem and then propose an image representation method that can capture both region structure and texture features, and a sparse Bayesian MIML algorithm which can produce probabilities indicating the confidence of annotation. The proposed algorithm framework is evaluated on a real clinical dataset containing 12,700 skin biopsy images. The results show that it is effective and prominent.

## 1. Introduction

Skin diseases are common in our daily life. Most of the skin diseases are not harmful to our health, while some kinds of them would lead to serious problems for our health. For example, malignant melanoma is a highly aggressive skin cancer which looks just like some harmless nevi in some cases. Pemphigus mostly characterized by the development of blisters on the skin is a rare skin disorder that leads to severe infection without effective treatment. Consequently, rapid recognition and correct diagnosis are important to the grave skin diseases as well as neoplasms, bullous dermatoses, sexually transmitted diseases (STD), and so forth. However, it is a great challenge for doctors specializing in dermatology since there are more than 3,000 kinds of diseases in this field, and what is worse is that the number of patients in dermatology is increasing rapidly [1], leading to great burden for doctors to precisely inspect large amount of cases every day.

Generally there are two categories of skin imaging inspection methods. The first is skin surface imaging. A doctor could be confident of making a diagnosis through observation and routine examination on the skin surface in some cases. However, in many other cases, especially in cases of skin cancer, a doctor is not easy to make a diagnosis decision when only skin surface information is available. The second is skin biopsy imaging, which is the imaging of slice of skin tissue under microscope. Skin biopsy images reflect the pathological changes behind skin lesions at a microscopic level. It is widely accepted that histopathology is the gold standard of diagnosing a skin disease [2]. Skin biopsy imaging can provide valuable information of what happens under skin surface. To reach correct annotation or diagnosis, a doctor needs not only professional knowledge and rich experience in inspecting skin lesions, but also deep understanding of skin histopathological imaging. While analyzing skin biopsy images consumes more time and requires more skills, differentiating normal/lesion regions or similar skin diseases becomes great challenges for doctors. Meanwhile, current skin biopsy image inspection is heavily relied on experience and professional knowledge of histopathological laboratory experts, which are subjective and unstable. To obtain a stable and reproducible diagnosis result, a computer-aid diagnosis (CAD) system is necessary.

**Figure 1:**
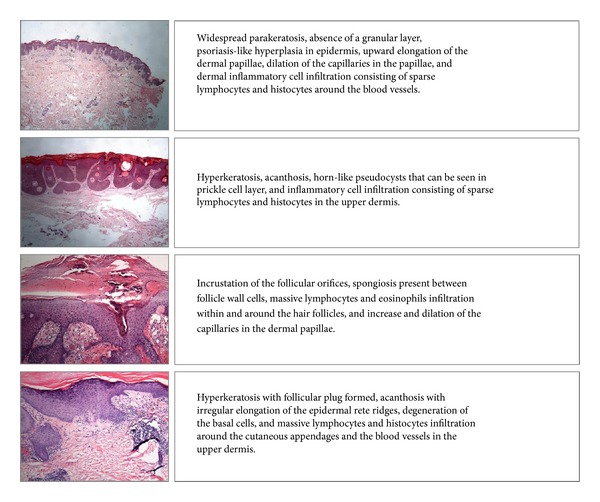
Example of skin biopsy images and their corresponding description in plain text.

**Figure 2:**
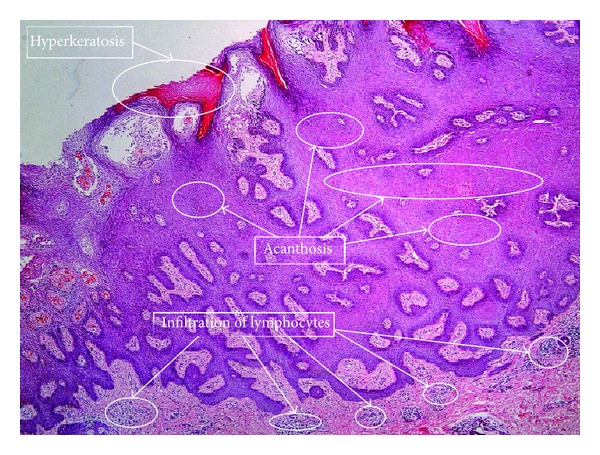
Dermatological terms and their corresponding regions.

Hence it is meaningful to develop computational methods for automatic feature recognition and annotation of skin biopsy images. However, there are some significant challenges due to the complex structures and textures of biopsy images and indirect relationship between historic diagnosis records and images. First of all, in dermatological practice, when annotating biopsy skin images, doctors only give plain text description for a patient attached to several skin biopsy images. The plain text description involves a set of standard dermatological annotation terms and some linked words to show key features reflected by the biopsy images, as shown in Figure 1. However, in fact, the dermatological terms only reflect certain local regions instead of the whole image. See Figure 2 for details. Only one or more small local regions is responsible for a certain dermatological term. However, the correspondence between dermatological terms and local regions is unknown in current datasets. Thus we cannot model this correspondence directly.

Another challenge is that, even for the same term, its corresponding local regions may be significantly varied in size, shape, texture, lightening, inner structure, or the relation between local regions with different terms. In addition, we should be aware of the fact that sublayers of a skin tissue are strictly ordered, leading to some correlations between local visual regions as well as the corresponding features [3]. All these challenges make the task more difficult to tackle compared with traditional machine learning ones.

Several attempts have been reported publicly to build models or classifiers for skin image automatic annotation or recognition. A portion of them have attempted to design different color space-based feature extraction methods and to apply different machine learning models to achieve good performance for different kinds of skin diseases [4–6]. However, a large amount of these methods have to face the problem of manually labeling lesion regions. In order to build a training dataset comprising both normal and lesion skin images, we are required to pick out normal and lesion regions for each skin image. Meanwhile, a large number of histopathological image analysis methods have also been reported for classification or grading of biopsy images [7–10]. But few of them attempted to model the indirect relationship between histopathological features and parts of a biopsy image.

Moreover, many previous methods required specialized knowledge to choose a proper color space representation and a model, which is not feasible in most cases. Recently, Bunte et al. [11] proposed a machine learning framework to combine several color space representation methods through a weighting procedure. Zhang et al. [12] proposed to convert the skin biopsy image feature recognition problem into a multi-instance (MI) learning problem and then solve it by current well-studied MI algorithms, which is the first attempt to tackle the skin biopsy image annotation problem within machine learning framework. In their paper, they applied a famous graph cutting algorithm, named nnormalized cut [13], to generate visual disjoint regions and then apply image feature extraction algorithm for each local region, so as to turn each image into a MI sample. However, they simply trained an individual MI learner for each target feature to be recognized, discarding the correlation between target features, which is not sufficient from a medical point of view.

In this paper, we attempt to tackle the skin biopsy image feature extraction problem under a recently proposed machine learning framework, multi-instance multilabel (MIML) learning. We first show that the problem is naturally a MIML learning problem. Then we propose a sparse Bayesian MIML learning algorithm with a Gaussian prior as the main model, which is able to model a posterior distribution of the target features giving images as input. We evaluate the proposed algorithm on a real dataset from the department of dermatology and venereology of a large local hospital. The evaluation results show that the proposed algorithm framework can effectively annotate the concerning terms of skin biopsy images superior to existing methods.

## 2. Materials and Methods

### 2.1. Materials

We aim at building a machine learning model for annotating a given skin biopsy image with a set of standard dermatology terms. The skin biopsy images are digitally stored. The size of each image is 2048 × 1536 pixels with 24*k* colored. The image files are fed to the model that outputs a binary vector to indicate whether the terms are annotated. We consider totally 15 annotation terms which appeared in the electronic records and regarded important for diagnosis in this study.  Table 1 lists 15 terms and their occurrence ratios in the whole evaluation dataset.

In our evaluation dataset, each patient has at least one skin biopsy image of the target skin tissue, associated with a plain text description given by an expert. We only select an image for each patient and assume that each selected image contains all terms in the text description. Then we can convert the text description into a binary vector through simple word-matching procedure. Thus the original problem becomes a multilabel binary classification problem.

We further formally define the problem as follows. Let *D* = {(*X*_1_, *T*_1_),…, (*X*_*n*_, *T*_*n*_), *X*_*i*_ ∈ *I*, *T*_*i*_ ∈ *W*} be a set of images associated with the annotated terms, where *X*_*i*_ is an image, *T*_*i*_ = {*t*_1_,…, *t*_*m*_*i*__} is a set of terms associated with the image, and *I*, *W* stand for the whole set of images and terms, respectively. The problem is to learn a function *f* : *I* → *W* with a training image set *D* such that when given a test image *X*_*t*_ it can give the posterior probability of each term in *W* to be annotated to *X*_*t*_.

To represent the key features of a given image, different feature extraction methods have been proposed and developed and in various fields of image understanding research [7]. However, a large body of feature extraction methods previously applied in histopathological image analysis, which extract global features, is not suitable for our biopsy image annotation task. Because in our problem there are *m* to *n* relationships between notation terms and local regions within images, methods extracting global features are not able to express local features corresponding to each region of interest.

If a given image can be cut properly to generate meaningful regions, the above correspondence can be directly modeled. The proper cutting of a given image should generate regions attached with terms as few as possible. Such regions are relatively simple and easy to be described. In histopathological image analysis, several image cutting methods have been applied in different tasks. Caicedo et al. [4] proposed a bag-of-words approach for histopathological image annotation. They divided an image into blocks of equal size to generate a codebook for feature representation. Ji et al. [14] and Li et al. [15] applied the almost same block-cutting method to generate MI samples from given images. Another region generating method that should be mentioned is based on block clustering proposed by Chen and Wang [16]. They generated regions by clustering 2D waveform transformation coefficiencies of each block. Thus similar blocks can be gathered into a single cluster. In their work clusters were regarded as regions and it generated discontiguous regions, not regions in common sense.

**Figure 3:**
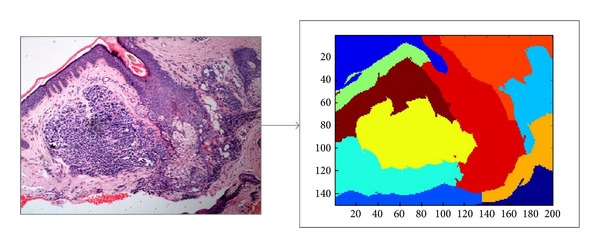
Normalized cut with *k* = 11.

However, such cutting approaches cannot generate regions of medical meaning as we need. As shown in our previous work [12], the model that is built upon such region generating methods cannot properly capture the direct medical knowledge and experience for annotating biopsy images. An experienced doctor would annotate an image by directly inspecting some local visual disjoint regions within the image. Following this observation, we apply the same idea to cut a given image into *k* visual disjoint regions through the normalized cut algorithm proposed by Shi and Malik [13]. The number of regions should be set before running the algorithm.  Figure 3 shows the result of normalized cut for an skin biopsy image with *k* = 11.

It should be noted that there is not any optimal *k* for the annotation problem, since the concept of local region is not an actual cutting of an image. A smaller *k* leads to larger regions, which may contain more than one term, while fragment regions may be generated if *k* is large. Hence we add a region size constraint when running the cutting algorithm. A generated region should contain at least 1500 pixels to avoid too much fragments, along with a relatively large *k*. Thus we can get as much as possible regions but avoiding too much fragments.

To further express each generated region as a vectorial representation, we propose a feature representation method that can capture both texture and structure features of regions. The method combined the features extracted through the method introduced in our previous work [8, 12] and features from a graph view of the image. Briefly saying, for the first part of the features, the method performs a waveform transformation for each equal-sized block within each region and combines the waveform transformation coefficiencies to form a 9-ary real vector for each region. To make the paper self-contained, we present some details of the extracted features. The first three features *f*_1_, *f*_2_, *f*_3_ are means of *L*, *U*, *V* values of all pixels within a region. The next three features *f*_4_, *f*_5_, *f*_6_ are mean DWT coefficients HH, HL and LH of all blocks. The last three features are the 1st, 2nd, and 3rd order normalized criteria [17] of the whole region.

**Figure 4:**
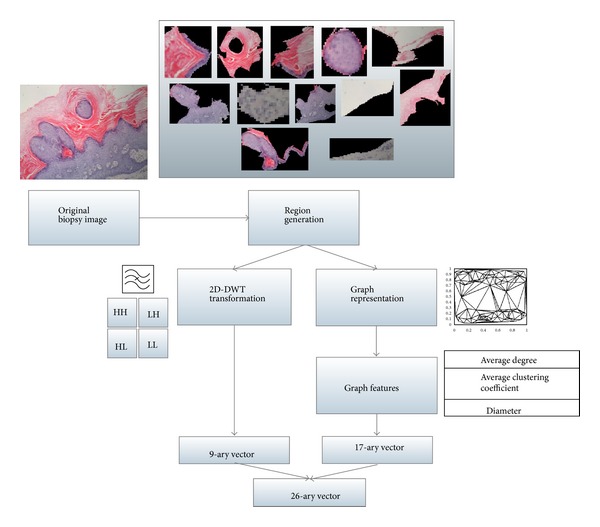
Feature extraction for local regions.

For the second part of the features, we represent a region as a graph in which nodes are centroids of clusters of pixels and edges are the relationship between nodes with real weights. We apply a heuristic algorithm [5] to seek the centroids of local similar pixels. Then a Delaunay triangulation method [18] is applied to the set of centroids to add edges. Graph representation methods are widely used in histopathological image analysis for it is able to capture the structure of a tissue [7, 9, 10, 19].  Figure 4 illustrates the main steps of our region feature extraction procedure.

There are three types of graph features considered in our feature representation. The first is average degree of nodes belonging to each cluster in the graph. It can be simply obtained by averaging the degrees of all nodes belonging to the same cluster. The degree of a node is the number of edges. The second is average clustering coefficient (ACC) [20], which measures the average connectivity of a node and its neighbors. The ACC for node *i* is defined as

(1) $$\begin{array}{c} {\text{ACC}}_{i}=\frac{2C_{i}}{d_{i}\left(d_{i}-1\right)}. \end{array}$$

In (1), *C*_*i*_ is the number of edges between node *i* and its neighbors and *d*_*i*_ is the degree of node *i*. The neighborhood between each pair of nodes is measured by the Euclidean distance. We calculate the values of ACC for nodes belonging to different clusters. We compute the average ACC of all nodes in the graph and nodes in the same cluster. Hence there are *p* + 1 average ACC where *p* is the number of clusters. The third is the diameter of the graph, which is defined as the shortest path of the longest path between pair of nodes on the graph. In our work *p* = 4, there are 4 average degrees, 4 × 3 different types of node connection, which results in 12 ACCs, and finally a diameter value of the whole graph. Totally we get a 17-ary feature vector.

**Figure 5:**
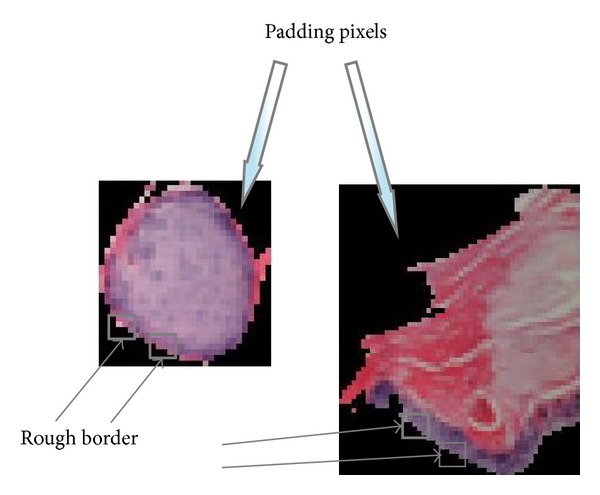
Padding pixels.

Since the generated regions are irregular in shape, padding pixels (in black) must be excluded from our feature extraction procedure. To do this, for the texture features, blocks that have at least one black pixel are discarded. Since the block in our method is of 4 × 4 pixels, it leads to a rough border of the original region which would not significantly affect the texture features. For graph features, it is not a problem since the black pixels would of course be clustered into a single cluster. Thus we can simply discard such black cluster to get rid of padding pixels. Details of the above idea were presented in our recent work [8].  Figure 5 illustrates the processing of padding pixels in our feature extraction procedure.

Thus, a skin biopsy image is decomposed into a MI example (bag), in which visual disjoint regions are instances. Moreover, we can define a binary vector to indicate whether an annotation term is associated with a given image. An annotation term can be regarded as a label associated with an image. Hence the biopsy image annotation problem can be naturally considered as a multi-instance multilabel (MIML) problem. Based on the relationship between regions and terms from clinical experience, we tackle the problem under the standard MI assumption which was firstly introduced by Dietterich et al. [21], assuming that a sample was labeled positively if at least one instance in it is positive and negative otherwise. The standard MI assumption has been widely used in bioinformatics study [22] and it is also suitable for this work.

### 2.2. Methods

#### 2.2.1. Sparse Bayesian MIML Learning Framework

In the previous subsection, we have shown that the problem is naturally a MIML problem. Now we propose a novel algorithm to solve this problem effectively. The general idea is that we first randomly construct a set of basic MIML learners and then learn a sparse weights vector under the relevant vector machine (RVM) [23] framework to combine the basic learners together. The learning framework prunes off many learners by automatically driving the corresponding weights to zero so as to get a sparse solution. The motivation of this work is the consideration of time complexity of building a good MIML learner. A weighted ensemble method is adopted, and the weights are determined by RVM method. The method does not require basic learners of good quality. It can find an optimal combination of learners of low quality at relatively low cost.

#### 2.2.2. Generating Basic Learners

We make use of a recently proposed Bayesian MIML learning model [24] for the generation of MIML basic learners. The method directly models a predictive distribution of terms conditioning on training data with a Gaussian process (GP) prior. We introduce a set of unobserved real-value functions *f* = {*f*_1_,…, *f*_*s*_} ranging from [0,1], where *s* is the number of target labels. The value of *f* for a given instance (region) indicates to which extent it should be annotated with the *s* concerning terms. Under the standard MI assumption, the bag label can be determined by a max⁡ or soft max⁡ function over *f*_*i*_ on all instances in the bag [25].

We formally describe the procedure of basic learner construction as follows. The goal is to model the predictive probability of the concerning annotation terms *T*, giving the training set *D*, a prior *K* ^GP^, and a test sample *x*, which can be expressed as *p*(*T* | *D*, *x*, *K* ^GP^). The prior *K* ^GP^ can be given by a kernel function through a Gaussian process. The likelihood function associated with latent functions *f* on *D* can be expressed as

(2) $$\begin{array}{c} p\left(T\;|\;F\right)=\overset{s}{\underset{i=1}{\prod }}\overset{n}{\underset{j=1}{\prod }}p\left(t_{i}\;|\;F_{ij}\right), \end{array}$$

where *F*_*ij*_ is the value of applying *f*_*i*_ to all instances in bag *x*_*j*_ and *F* is a matrix containing all values of applying all *f* on *D*.

Since *F* is unknown, we impose a prior for *F* to avoid overfitting when evaluating it. Following Bonilla et al.'s work [26], a Gaussian prior for *F* with zero mean and covariance is defined as follows:

(3) $$\begin{array}{c} p\left(F\right)=N\left(F\;|\;0,K^{\text{GP}}⊗K\right). \end{array}$$

In (3), *K* stands for the gram matrix for some kernel functions (e.g., RBF or poly kernel) in instance space and *K* ^GP^ in fact indicates the relationship between terms to be annotated. In [24], they adopted a marginal likelihood maximization method to find the optimal *K* ^GP^, which is expensive. In this work, we do not directly work out the optimal solution for *K* ^GP^. On the contrary, we randomly generate *K* ^GP^ *Q* times and then learn a vector of weights to obtain an optimal combination.

With *K* ^GP^, we can further derive the posterior distribution given a training dataset *D* as

(4) $$\begin{array}{c} p\left(F\;|\;D,T\right)=\frac{p\left(T\;|\;F\right)p\left(F\right)}{\int p\left(T\;|\;F\right)p\left(F\right)dF}. \end{array}$$

Notice that the second *p*(*T*) = ∫*p*(*T* | *F*)*p*(*F*)*dF* is a constant value since *T* is constant and *F* is integrated out. Thus it can be ignored. Because *p*(*T* | *F*)*p*(*F*) is not a Gaussian [26], we use some approximation methods to evaluate it. Following Nickisch and Rasmussen's work [27], we apply the Laplace approximation to convert *p*(*T* | *F*) into a Gaussian near its true mode. According to [26, 27], we can directly write down the mean and variance of the approximation distribution for *p*(*T* | *F*). Meanwhile we notice that *p*(*F*) is also a Gaussian, which leads to a Gaussian distribution for *p*(*F* | *D*, *T*).

The predictive probability can then be derived from the likelihood, prior, and posterior distribution aforementioned. We have

(5) $$\begin{array}{c} p\left(t_{i}\;|\;D,T,x\right)=\int \max\left(F_{x}\right)p\left(F_{x}\;|\;D,T,x\right)dF_{x}, \end{array}$$

where *x* is a test bag (image) and *F*_*x*_ is a vector of applying all *f* to all instances in *x*. The first term on the right-hand side reflects the standard MI assumption, meaning that the largest value among *f* determines the probability to be annotated with the corresponding term. For computational convenience, we often use soft max⁡ function instead of max⁡ in (5), given by ln⁡∑_*i*_ *e* ^*a*_*i*_^. The predictive distribution is also a Gaussian and can be solved directly as follows:

(6) $$\begin{array}{c} p\left(t_{i}=\text{true}\;|\;D,T,x\right)=\int \ln\left(\frac{\sum_{j}^{}F_{xj}}{\left|F_{x}\right|}\right)p\left(F_{x}\;|\;D,T,x\right)dF_{x}. \end{array}$$

The right-hand side of (6) is a Gaussian, which can be determined through a EM-like procedure [27]. An important thing should be noticed is that (6) has a parameter matrix *K* ^GP^ that controls the relationship between terms.

The time complexity of the above procedure can be analysed as follows. Suppose we generate a set of *Q* basic learners and |*T*| annotation terms. For each learner, there is a random sampling procedure for *K* ^GP^ which requires *O*(|*T*|^2^) operations; training a MIML learner requires *O*(|*T* | ×|*D*|^2^), where |*D*| denotes the number of instances in training dataset.

#### 2.2.3. Sparse Bayesian Ensemble

Since the cost of calculating the optimal *K* ^GP^ is very high, we randomly set them *Q* times to obtain a set of different learners and then apply a weighted ensemble procedure as follows:

(7) $$\begin{array}{c} f_{\text{ens}}\left(x\right)=\overset{Q}{\underset{i=1}{\sum }}f_{i}\left(x\right). \end{array}$$

**Figure 6:**
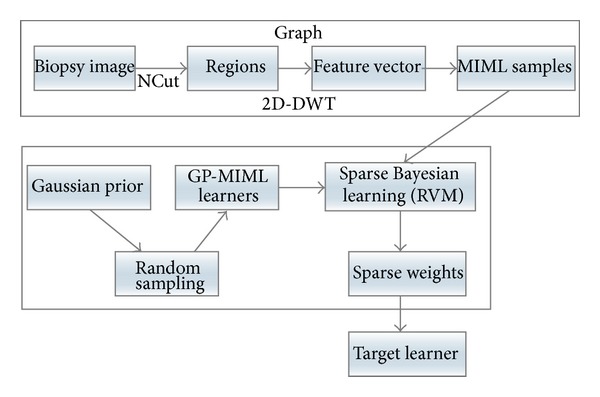
Main steps of the proposed algorithm.

A RVM-like algorithm [23] is adopted to find the optimal weights to combine them. The main reason for using RVM is twofold. On one hand it is purely based on Bayesian theory which is consistent with our basis learner. On the other hand, RVM can give a sparse solution which is preferred in large data analysis and fast annotation.  Figure 6 shows the main steps of the proposed algorithm framework.

The target model is a weighted ensemble of a set of basic learners. To get a sparse representation, we impose an ARD prior [28] on the weights *w* which is a Gaussian with zero mean and different variances *α*_*i*_ for each weight *w*_*i*_. In RVM's optimization procedure [23], a large body of variances would be driven to infinity leading the corresponding weights to zero. Hence a large body of weights would be pruned off from the model and final a sparse model is obtained. Formally, let *w* = {*w*_1_,…, *w*_*Q*_} be a set of weights associated with *Q* learners. A Gaussian prior with zero mean and different variances is imposed on *w*. Tipping's work [23] indicated that when applying a maximum a posterior (MAP) learner to learn an optimal *w*, a large body of *w* would be driven to zero. Following this idea, we apply RVM algorithm on *w* given the training dataset *D*.

Please note that the weighted ensemble may not follow a Gaussian distribution. This is because ∑_*i*_ *w*_*i*_ is not guaranteed to be 1. A normalization procedure should be applied to obtain a normalized combination

(8) $$\begin{array}{c} w_{i}=\frac{w_{i}}{\sum_{j}^{}w_{j}}. \end{array}$$

By applying RVM, a smooth learner can be obtained which captures the general features of the whole training dataset. RVM adopts an iterative procedure to find optimal weights.

## 3. Results and Discussion

### 3.1. Results

We present the evaluation result of the proposed algorithm on a real dataset gathered from a large local hospital. The setting of basic learner generation is the same as [24] and the setting of RVM follows Tipping's original implementation [23]. The proposed method is compared with some existing approaches in histopathological image analysis. Since some of them are not consistent with the MIML setting in our work, we would implement them on a more general foundation for image analysis.

#### 3.1.1. Dataset and Data Preprocessing

The evaluation was carried out on a real skin disease clinical dataset from a large local hospital. The dataset has been reconstructed to get rid of irregular patient information and low quality biopsy images.

The biopsy images in the evaluation dataset are taken by a Leica DFC290 digital camera with 20x, 40x, and 100x microscope objective lenses. The images are taken in RGB color space and stored in JPEG format. For convenience, we only keep images at 40x magnification ratio. It contains 4,123 patients with 12,700 images. The images are 2048 × 1536 pixels with 24*k* colors. For computational efficacy, they are rescaled to 800 × 600 pixels. There are three 40x biopsy images for each patient on average. We consider 15 features to be annotated, corresponding to 15 standard terms, as shown in Table 1, and then convert the plain-text description into a 15-ary binary vector in which each element indicates whether the corresponding term exists in the diagnosis record in plain text, as shown in Figure 1. Since most doctors use standard terms and link words in their description, training dataset of good quality can be obtained in this way.

Each image associated with a patient is converted into a bag through normalized cut and then a feature extraction method combined with waveform transformation and graph representation. For normalized cut, the number of regions *k* must be set manually. In our evaluation we set *k* = 11 which means an image would be converted into a bag consisting of 11 instances. A further discussion on the setting strategy of *k* is presented in the next section. Different images of the same patient are associated with the 15-ary binary of the patient. We denote the dataset generated through the above procedure as *D*1. For waveform transformation, each region should be divided into blocks of size 4 × 4 pixels. Blocks containing at least one black pixel would be discarded. For graph representation, the number of clusters *p* is set to 5, assuming that there are 5 different tissues in each image on average. In node identification algorithm, circles containing less than 20 pixels would not be taken into account.

Since there are other compared methods that are not consistent with the MI setting, we generate another three data representations, namely, *D*2, *D*3, and *D*4, for these methods. Data representation *D*2 is based on the equal-sized block cutting method proposed in [4]. We first cut each image into 4 × 4 blocks and apply a scale-invariant feature transform (SIFT) descriptor [29] to extract features and use histogram to express it as a feature vector. *D*2 is a bag-of-words [14] image representation which is widely used in image understanding. Dataset *D*3 is an equal-sized block MI sample representation proposed in [15]. The main procedure is similar to *D*2, but it directly regards each block with SIFT representation as an instance. Hence in *D*3 there are totally 30,000 instances in each bag. Finally dataset *D*4 is a clustering based representation. It clusters equal-sized blocks represented in real value vector and regards each cluster as an instance. Details of this method can be found in [16].  Table 2 lists the above data representation and their consistent methods for comparison.

Note that these datasets are only different in their preprocessing steps. In Table 2 we can see that method *M*4 can be fed with *D*1 and *D*3, for *M*4 is a MIML learning algorithm naturally consistent with MI data representation. However *D*3 cannot be fed to *M*1 because the idea of *M*1 is to regard each visual disjoint region instead of equal-sized block, as an instance. Different definitions of instance are originated from the difference of underlying idea of the problem. A single block may not contain medically acceptable features, which is not consistent with our MI framework.

#### 3.1.2. Evaluation Criteria

We adopt five different criteria to evaluate the performance of the proposed method and the compared methods. The first is accuracy, a zero-one loss function evaluating whether a single term is correctly annotated. It can be applied to evaluate the performance of methods that annotate only one term each time. Since the proposed method is a MIML one, it can be regarded as a multilabel learner. Several evaluation criteria have been proposed in multilabel learning and MIML learning study [30]. Introducing such criteria is necessary for our evaluation. Formal definition of the four multilabel evaluation criteria can be found in [30].  Table 3 lists five criteria used in our evaluation.

#### 3.1.3. Evaluation Result

For the methods shown in Table 2, we use the same setting for evaluation. The evaluation is launched through a supervised learning manner. The whole dataset (with 12,700 images) is divided into training set and test set at a ratio 3 : 7. To avoid learning bias, the occurrence ratios of the concerning terms in Table 1 were kept the same as the training set. For method *M*1, we use a modified GPMIL and RVM implementation which were originally proposed by Kim et al. [31] and Tipping [23].

The first evaluation focuses on the annotation accuracy. Recall that we have 15 concerning annotation terms.  Table 4 gives the results of annotating each term by different methods in Table 2.

**Figure 7:**
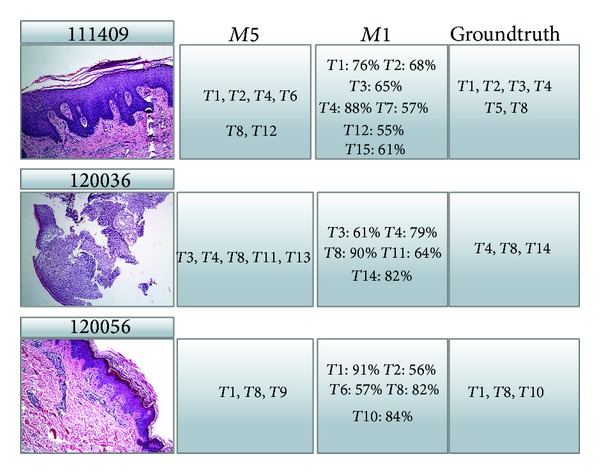
Sample outputs of methods *M*1 and *M*5.

It should be noted that the output of method *M*1 is a 15-ary real vector indicating the probabilities of annotating 15 terms. In this part of evaluation, we simply use an indicator function which outputs 1 if the probability is not less than 0.5 and 0 otherwise.  Figure 7 shows some outputs of *M*1 and *M*5, in which the probabilities of the concerning terms are shown, as well as the groundtruth annotation terms.

For each row in Table 4, the best accuracy is highlighted. It can be seen that *M*1 achieved the best performance in annotating most terms, which shows the effectiveness of our method. However, for some terms, for example *T*4 and *T*5, method *M*4 performed better than *M*1 and *M*2. We think this is because our graph cutting representation is not consistent with these terms, while the more general grid cutting representation is better.

**Figure 8:**
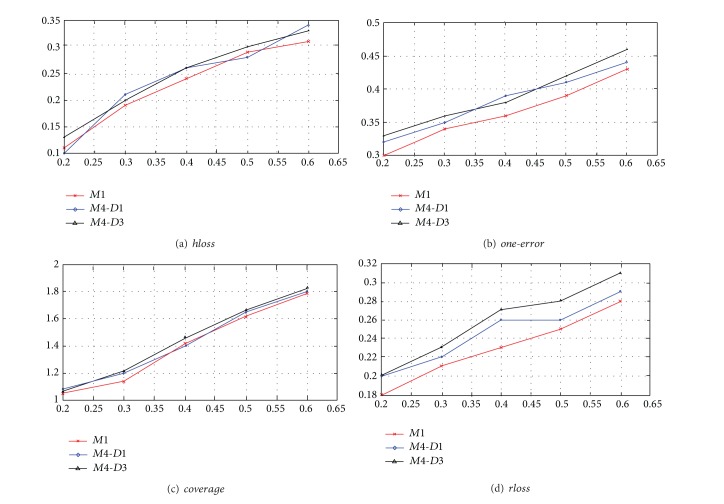
Evaluation result of four criteria.

The second evaluation focuses on the performance of annotating several terms simultaneously. Note that, in previous part of evaluation, accuracy of annotation was evaluated term by term; hence the overall accuracy of annotating all concerning terms may not be as high as the individual ones. We adopt four criteria listed in Table 3 to show the performance of annotation of all terms at the same time. Some criteria rely on the ranking of terms. We can get a natural ranking for the proposed method since it gives the probabilities for all terms. For other methods to be compared in our evaluation, we use the ranking strategy similar to [30]. Note that methods *M*2, *M*3, and *M*5 are not multilabel classifiers. Hence we only compare *M*1, *M*4 with *D*1 and *M*4 with *D*3.  Figure 8 shows the performance evaluated by the above four criteria.

According to Table 3, the smaller results of the four criteria indicate the better performance. From Figure 8, it can be seen that method *M*1 achieved best performance compared to other methods in a multilabel classification setting at different training data ratios. For method *M*4, different data representations *D*1 and *D*3 lead to different performances. It can be seen that *D*1 is better than *D*3 in most cases. Since the intuition of *D*1 and *D*3 is totally different, it may be concluded that the representation *D*1 is more consistent with the term set and the models.

**Figure 9:**
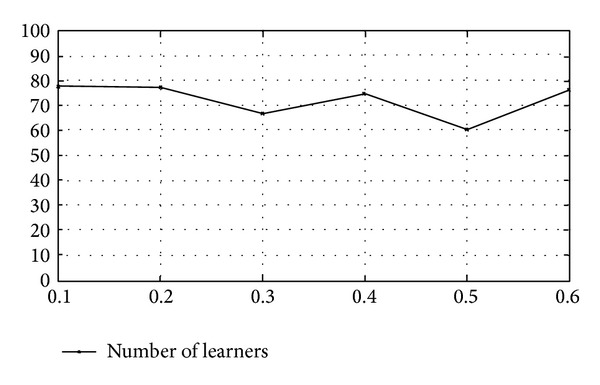
Sparsity and number of basic learners.

Finally we evaluate the sparsity of the proposed model. We vary the ratios between training data and test data and plot them with the nonzero-weighted basic learners after RVM procedure. In this case the set of basic learners contains 200 learners; that is, *Q* = 200.  Figure 9 shows the result.

**Figure 10:**
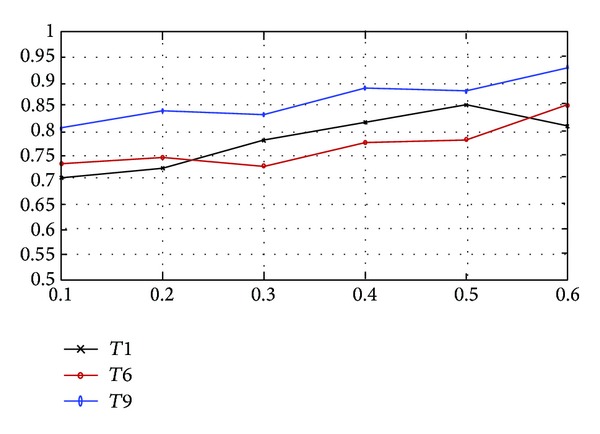
Accuracy and the size of training set.

From Figure 9 we can see that RVM procedure can prune off about 2/3 learners, which yields a sparse ensemble learner.  Figure 10 shows the corresponding annotation accuracy of different training set sizes. It can be seen that large training set would lead to high accuracy.  Figure 9 indicates that the number of nonzero-weighted learners is stable at different training set sizes. The performance of the proposed method obeys the basic principle of machine learning; that is, more training data means model of high accuracy. For illustration, Figure 10 shows the relationship between accuracy and the size of training set for terms *T*1, *T*6, and *T*9.

### 3.2. Discussions

Some important issues are worth addressing here. First, we must answer why MIML rather than MI framework is consistent with our task. MIML learning problem can be decomposed into several MI learning problems if we assume labels are independent of each other. When coming to our annotation problem, it is observed that there are correlations between annotation terms, including the cooccurence of some terms or the absence of other terms. Furthermore, some annotation terms may appear at the same time for some diseases. To capture the correlations mentioned above, MI learning framework which regards each annotation term independently is not sufficient. However, MIML learning framework is able to capture the relationship between annotation terms, as well as regions, which is superior to MI framework.

**Figure 11:**
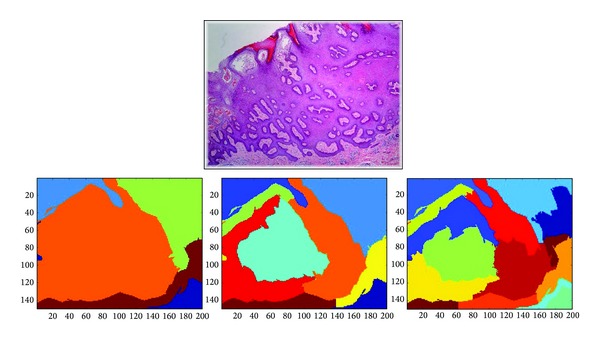
The result of normalized cut with different settings of *k*.

Second, our proposed regions generating method is based on normalized cut, which generates visual disjoint regions for a given image. The number of regions generated by normalized cut must be manually set. A small *k* would lead to large regions that may contain different terms. A large *k* would lead to fragment regions associated with the same term, as shown in Figure 11. However, in either case, MIML learning framework works according to the standard MI assumption [21, 32]. The former case is equivalent to an instance corresponding to more than one term. The latter case is equivalent to several instances corresponding to the same term. Though the quantity of *k* would not affect the effectiveness of MIML, too small *k* would affect the effect of feature extraction. A region contains different terms cannot be expressed as a real feature vector distinguishing between each term at the same time. Hence, in our work, we use a relative large *k* according to medical experience to avoid a region containing more than one term and too much fragments.

Third, a Bayesian model can generate probability for each concerning annotation term, which makes it available to build a more powerful model for automated skin disease diagnosis. Annotation terms can be regarded as latent variables between skin biopsy images and diseases, meaning that *p*(*w* | *I*) = ∑_*t*∈*T*_ *p*(*w* | *t*)*p*(*t* | *I*) for independent and identically distributed (i.i.d.) terms, where *I*, *t*, *T*, respectively, stand for diseases, images, a certain term, and the set of terms. And for non-i.i.d. terms, we can separate the terms into dependent term groups and apply almost the same equation as in the i.i.d. case. The method proposed in this paper can effectively evaluate *p*(*t* | *I*), and *p*(*w* | *t*) can be obtained directly from clinical experience. Hence, it is meaningful in CAD system design and implementation.

Finally, we discuss the multi-instance assumption implied in this work. We use the standard MI assumption [21] when considering the relationship between regions and terms. The standard MI assumption does not directly consider the impact of the number of regions and the relationship between regions to the terms. From clinical observation, most annotation terms can mainly be determined by a single region if the generated regions are not too small. Large region may contain more than one term, but it is also consistent with the standard MI assumption and this can be solved due to the power of MIML models. Though our proposed MIML model in fact considers such relationship, a simple assumption of the problem may lead to simple model.

## 4. Conclusions

In this paper we propose a MIML framework for skin biopsy image annotation. We adopt a famous graph cutting algorithm named normalized cut to transfer a biopsy image into a MI sample, in which each region is regarded as an instance. To effectively express features of biopsy images, each region is expressed as a 9-ary real vector. To reduce the model complexity and training time, we propose a novel sparse Bayesian MIML learning model, which applies a RVM-like algorithm to obtain a sparse weighted combination for a set of basic learners. We also make use of the well-studied Bayesian MIML learner as basic learners. Evaluation of a real clinical dataset shows that the proposed model can achieve good performance and reach a medical acceptable result. We have achieved an annotation accuracy up to 85% in our evaluation dataset.

The proposed annotation framework directly models doctor's experience of annotation biopsy images. Different from previous work, it is explicable since it can give the correspondence between local visual disjoint regions and the terms associated with them. Future work will focus on studying the relationship between biopsy images and the final diagnosis given the annotation term set as latent variables. And the feature fusion algorithm towards an effective feature representation is another research direction.

## Figures and Tables

**Table 1:** 15 considered annotation terms and their occurence frequency.

Number	Name	Rate
*T*1	Retraction space	28.65%
*T*2	Papillomatosis	22.71%
*T*3	Follicular plug	1.8%
*T*4	Hypergranulosis	32.15%
*T*5	Horn cyst	4.14%
*T*6	Basal cell liquefaction degeneration	6.48%
*T*7	Thin prickle cell layer	2.61%
*T*8	Infiltration of lymphocytes	9.12%
*T*9	Hyperpigmentation of Basal cell layer	36.99%
*T*10	Nevocytic nests	18.56%
*T*11	Munro microabscess	7.72%
*T*12	Acanthosis	19.05%
*T*13	Absent granular cell layer	23.24%
*T*14	Parakeratosis	6.81%
*T*15	Hyperkeratosis	11.30%

**Table 2:** Data representation and their consistent methods.

Method	Reference	Dataset
*M*1: our method	This work	*D*1
*M*2: MIBiopsy	Zhang et al. [12]	*D*1
*M*3: bag of features	Caicedo et al. [4]	*D*2
*M*4: MIMLSVM	Li et al. [15]	*D*1, *D*3
*M*5: DDSVM	Chen and Wang [16]	*D*4

**Table 3:** Evaluation criteria for multilabel learning.

Name	Equation
*hloss*	Evaluate the number of misclassified label pairs

*one-error *	Evaluate the portion that a label of highest probability is not a correct label

*coverage*	Evaluate the average distance to go down to find the proper label for a given image

*rloss*	Evaluate the average fraction of label pair that are misordered in the ranking list

**Table 4:** Annotation result evaluated by accuracy.

Term	*M*1	*M*2	*M*3	*M*4	*M*5
*T*1	**78.2%**	76.1%	70.6%	75.9%	68.3%
*T*2	**80.3%**	75.9%	76.1%	74.5%	73.8%
*T*3	77.7%	**79.5%**	77.8%	76.2%	68.5%
*T*4	81.3%	81.2%	80.5%	**82.4%**	81.2%
*T*5	69.3%	66.5%	67.9%	**70.1%**	67.4%
*T*6	**76.3%**	75.0%	71.7%	74.2%	72.3%
*T*7	**77.8%**	77.4%	76.5%	75.8%	75.9%
*T*8	85.1%	**85.2%**	84.6%	83.8%	80.9%
*T*9	**87.3%**	86.8%	81.4%	83.0%	78.2%
*T*10	**75.9%**	75.4%	74.5%	73.8%	72.0%
*T*11	69.9%	**71.5%**	68.9%	70.7%	69.6%
*T*12	**78.0%**	76.1%	73.2%	75.8%	73.2%
*T*13	79.2%	**80.1%**	77.2%	78.8%	72.5%
*T*14	80.6%	81.2	77.2%	**81.9%**	73.5%
*T*15	**87.9%**	86.4%	82.6%	83.1%	80.2%

## References

[1] Sellheyer K, Bergfeld WF (2005). A retrospective biopsy study of the clinical diagnostic accuracy of common skin diseases by different specialties compared with dermatology. *Journal of the American Academy of Dermatology*.

[2] Fogelberg A, Ioffreda M, Helm KF (2004). The utility of digital clinical photographs in dermatopathology. *Journal of Cutaneous Medicine and Surgery*.

[3] Fernandez DC, Bhargava R, Hewitt SM, Levin IW (2005). Infrared spectroscopic imaging for histopathologic recognition. *Nature Biotechnology*.

[4] Caicedo JC, Cruz-Roa A, González FA, Combi C, Shahar Y, Abu-Hanna A Histopathology image classification using bag of features and
kernel functions.

[5] Tosun AB, Kandemir M, Sokmensuer C, Gunduz-Demir C (2009). Object-oriented texture analysis for the unsupervised segmentation of biopsy images for cancer detection. *Pattern Recognition*.

[6] Sertel O, Kong J, Catalyurek UV, Lozanski G, Saltz JH, Gurcan MN (2009). Histopathological image analysis using model-based intermediate representations and color texture: follicular lymphoma grading. *Journal of Signal Processing Systems*.

[7] Ozdemir E, Gunduz-Demir C (2013). A hybrid classification model for digital pathology using structural and statistical pattern recognition. *IEEE Transactions on Medical Imaging*.

[8] Zhang G, Yin J, Li Z, Su X, Li G, Zhang H (2013). Automated skin biopsy histopathological image annotation using multi-instance representation and learning. *BMC Medical Genomics*.

[9] Altunbay D, Cigir C, Sokmensuer C, Gunduz-Demir C (2010). Color graphs for automated cancer diagnosis and grading. *IEEE Transactions on Biomedical Engineering*.

[10] Gunduz-Demir C, Kandemir M, Tosun AB, Sokmensuer C (2010). Automatic segmentation of colon glands using object-graphs. *Medical Image Analysis*.

[11] Bunte K, Biehl M, Jonkman MF, Petkov N (2011). Learning effective color features for content based image retrieval in dermatology. *Pattern Recognition*.

[12] Zhang G, Shu X, Liang Z, Liang Y, Chen S, Yin J Multi-instance learning for skin biopsy image features recognition.

[13] Shi J, Malik J (2000). Normalized cuts and image segmentation. *IEEE Transactions on Pattern Analysis and Machine Intelligence*.

[14] Ji S, Li Y-X, Zhou Z-H, Kumar S, Ye J (2009). A bag-of-words approach for *Drosophila* gene expression pattern annotation. *BMC Bioinformatics*.

[15] Li Y-X, Ji S, Kumar S, Ye J, Zhou Z-H (2012). *Drosophila* gene expression pattern annotation through multi-instance multi-label learning. *IEEE/ACM Transactions on Computational Biology and Bioinformatics*.

[16] Chen Y, Wang JZ (2004). Image categorization by learning and reasoning with regions. *The Journal of Machine Learning Research*.

[17] Gersho A (1979). Asymptotically optimal block quantization. *IEEE Transactions on Information Theory*.

[18] Barber CB, Dobkin DP, Huhdanpaa H (1996). The quickhull algorithm for convex hulls. *ACM Transactions on Mathematical Software*.

[19] Demir C, Gultekin SH, Yener B (2005). Augmented cell-graphs for automated cancer diagnosis. *Bioinformatics*.

[20] Dorogovtsev SN, Mendes JFF (2002). Evolution of networks. *Advances in Physics*.

[21] Dietterich TG, Lathrop RH, Lozano-Pérez T (1997). Solving the multiple instance problem with axis-parallel rectangles. *Artificial Intelligence*.

[22] Wang X, Li GZ (2013). Multilabel learning via random label selection for protein subcellular multilocations prediction. *IEEE/ACM Transactions on Computational Biology and Bioinformatics*.

[23] Tipping ME (2001). Sparse Bayesian learning and the relevance vector machine. *Journal of Machine Learning Research*.

[24] He J, Gu H, Wang Z (2012). Bayesian multi-instance multi-label learning using Gaussian process prior. *Machine Learning*.

[25] Zhang M-L Generalized multi-instance learning: problems, algorithms and data sets.

[26] Bonilla EV, Chai KM, Williams CKI, Platt JC, Koller D, Singer Y, Roweis S (2008). Multi-task Gaussian process prediction. *Advances in Neural Information Processing Systems 20*.

[27] Rasmussen CE, Williams C (2006). *Gaussian Processes for Machine Learning*.

[28] Neal RM (1996). *Bayesian Learning for Neural Networks*.

[29] Lowe DG (2004). Distinctive image features from scale-invariant keypoints. *International Journal of Computer Vision*.

[30] Schapire RE, Singer Y (2000). BoosTexter: a boosting-based system for text categorization. *Machine Learning*.

[31] Kim M, de la Torre F, Fürnkranz J, Omnipress JT Gaussian processes multiple instance learning.

[32] Foulds J, Frank E (2010). A review of multi-instance learning assumptions. *Knowledge Engineering Review*.

